# Stability analysis of the coexistence equilibrium of a balanced metapopulation model

**DOI:** 10.1038/s41598-021-93438-8

**Published:** 2021-07-08

**Authors:** Shodhan Rao, Nathan Muyinda, Bernard De Baets

**Affiliations:** 1 KERMIT, Department of Data Analysis and Mathematical Modelling, Ghent University, Coupure links 653, 9000 Gent, Belgium; 2 Ghent University Global Campus, 119 Songdomunhwa-Ro, Yeonsu-Gu, Incheon, South Korea

**Keywords:** Ecological modelling, Theoretical ecology, Applied mathematics

## Abstract

We analyze the stability of a unique coexistence equilibrium point of a system of ordinary differential equations (ODE system) modelling the dynamics of a metapopulation, more specifically, a set of local populations inhabiting discrete habitat patches that are connected to one another through dispersal or migration. We assume that the inter-patch migrations are detailed balanced and that the patches are identical with intra-patch dynamics governed by a mean-field ODE system with a coexistence equilibrium. By making use of an appropriate Lyapunov function coupled with LaSalle’s invariance principle, we are able to show that the coexistence equilibrium point within each patch is locally asymptotically stable if the inter-patch dispersal network is heterogeneous, whereas it is neutrally stable in the case of a homogeneous network. These results provide a mathematical proof confirming the existing numerical simulations and broaden the range of networks for which they are valid.

## Introduction

One of the central questions in community ecology is why there are so many different species of plants and animals coexisting in nature^1,2^. This coexistence of large numbers of species has long been considered a paradox, a classical example being ‘the paradox of the plankton’^3^ in which many species of plankton coexist in the same ecological niche. This has motivated many community ecologists to try to solve the problem of species coexistence, often beginning with the premise that all individuals of all species live in a well-mixed, homogeneous, non-spatial habitat^4,5^. However, spatial heterogeneity (i.e., differences between populations and individuals at different geographical locations) is one of the most obvious features of the natural world. In addition, heterogeneities in the spatial landscape have been shown to have profound effects on the dynamics of populations and the structure of communities^6,7^. Thus, the study of spatial effects on community structure has more recently become a central theme in ecology^8^.

Spatial structure and dynamics determine the way the interactions among species living within a given habitat are interconnected^9^ to form the complex networks that characterize ecological communities. These complex networks can take many structural forms ranging from linear hierarchies to intransitive tournaments, which describe interactions where there is no single best competitor, but rather the network involves at least one 3-cycle (or intransitive triad)^10^. This structural form determines whether an ecological community is able to thrive and persist or will go extinct as it evolves over time^11^. For example, a linear hierarchy associated with resources critical for a species’ survival undermine coexistence because at equilibrium the dominant competitor will exclude all others^12^. On the other hand, theoretical and experimental studies have shown that cyclic competition combined with dispersal of species through space may facilitate the maintenance of diversity in a community due to the inability of one species to exclude all the others^13^. Such cyclic competition, analogous to the classical rock–paper–scissors (RPS) game, have been found to exist in a variety of real ecosystems including plant systems^14,15^, marine benthic systems^16^ and microbial populations^17,18^. Cyclic competition also plays a role in the mating strategy of side-blotched lizards^19^, the overgrowth of marine sessile organisms^20^, competition between mutant strains of yeast^21^ and in explaining the oscillating frequency of lemming populations^22^.

Non-transitive competition introduces interesting and complicated dynamics in time and space^23^. Early theoretical studies on cyclic competition among three species are based on the Lotka–Volterra model of ordinary differential equations (ODEs), which ignores the effects of the spatial domain and predicts a solution of unstable periodic dynamics that leads to the extinction of two of the species after a short transient^24,25^. However, numerous theoretical models have shown that the three competing species can coexist indefinitely if ecological processes such as dispersal, migration and cyclic competitive interactions occur over small spatial scales^17,26^. Space therefore plays a central role in mediating ecological dynamics and the use of spatial models in ecology has grown enormously over the last two decades.

Various models have been used to explore the effects of spatial structure on population dynamics. However, the most fundamental distinction between the different models is the way in which the spatial dimension is represented. Some models treat space explicitly, giving some sort of description at each spatial location at any given time, or implicitly, incorporating parameters that vary with spatial scale or following only the percent cover of different species across the landscape^7,27,28^. Models that treat space explicitly can be classified according to whether population sizes, space, and time are treated as discrete or continuous entities. Among these, continuous space reaction–diffusion partial differential equations^7^, metapopulation patch models^29^ and individual-based cellular automata^30,31^ are the principal approaches that have been used in studies of spatially structured populations. Our focus in this paper is on the metapopulation framework.

Metapopulation theory constitutes a useful framework for explicitly incorporating spatial effects in models for population dynamics in ecology. Whereas classical population models treat local populations as closed systems (no immigration or emigration), this assumption is probably not valid for many species. Movement of individuals among populations is common and can have profound effects on the dynamics of local populations. Metapopulation models describe an open system in which extinction and persistence of local populations depend on the movement of individuals among a set of habitat patches. This concept, introduced by Levins^32^ in 1969 to describe the population dynamics of insect pests in farmlands, has been broadly used in conjunction with networks to assess the viability of populations that persist in fragmented landscapes linked by dispersal and in modelling large-scale spatial transmission of emerging diseases.

Thus, a metapopulation consists of local populations, living in spatially discrete habitats (patches)—where population dynamics takes place and homogeneous mixing of individuals is assumed—connected to each other through dispersal or migration^33^. Many species often inhabit discrete areas of the landscape (ponds, woodlands in agricultural landscapes, and so on) where demographic processes occur within patches, and dispersal occurs between them. The movement of organisms between patches leads to local differences in colonization and extinction rates, which can influence the spatial distribution of a species over time^9,34^.

In typical metapopulation modelling, the combination of habitat patches and their connectivity (number of dispersal links from one patch to other patches) can be seen as a network (or directed graph), where patches represent the nodes of the network and dispersal routes represent the edges. Each edge of the graph is assigned a nonnegative constant known as the rate constant of migration. In^35^, the authors assume that migrations between patches are random with the migration rate constant being equal to the reciprocal of the number of dispersal links from a given patch to the other patches. They therefore define a dispersal graph to be homogeneous if all nodes (patches) have the same number of links, otherwise the graph is considered heterogeneous.

For many ecologists, the central question is how the structure or connectivity pattern of the underlying graph influences the dynamics of the metapopulation. Many model-based and empirical studies have shown that the persistence of metapopulations is influenced by connections between habitat patches^36^. For example, numerical simulations by Nagatani et al.^35^ have shown that in a metapopulation model for the rock–paper–scissors (RPS) game, the dynamics are significantly different when the dispersal graph is homogeneous compared to when the graph is heterogeneous. Specifically, their simulations show that the coexistence equilibrium within each patch is asymptotically stable in the case of a heterogeneous graph, while the same equilibrium remains neutrally stable in the case of a homogeneous graph (as in the single patch case). That is, heterogeneity leads to the indefinite coexistence of all three species with abundances equal to the coexistence equilibrium values, whereas homogeneity leads to the perpetual coexistence of all three species with periodically oscillating abundances (i.e., there exists a limit cycle around the coexistence equilibrium to which the trajectories converge). However, as far as we know, these numerical results have been provided without mathematical proof.

Here, we show, mathematically, that the numerical observations of^35^ are not only valid for RPS competition systems, but also extend to a broader class of competition networks. We first broaden the class of competition networks under consideration by giving a more general definition of homogeneity/heterogeneity that is based on the adjacency matrix of the dispersal graph without imposing any restrictions on the values of the migration rate constants. Then by combining concepts and results from game theory, chemical reaction network theory (CRNT) and dynamical systems, we provide a mathematical proof for the numerical observations in^35^, generalizing the scope of application to cover this broader class of competition networks within the patches. Specifically, we first consider a mean-field ODE model that describes the intra-patch dynamics in the absence of migration. The equilibrium point of this mean-field model is closely related to the Nash equilibrium (NE) of a symmetric zero-sum game and thus by analyzing the NE of the game, the behaviour of the coexistence equilibrium within each patch can be deduced. Then we borrow from CRNT the concept of detailed-balancedness^37,38^, a key feature for the kind of metapopulation models used in^35^, but not made explicit in it. We define a metapopulation model to be detailed-balanced if there exist positive equilibrium species proportions (or relative abundances) for which the overall migration rate of each species between any two patches is zero. Then, by assuming that our metapopulation model is detailed-balanced, we provide a mathematical proof for the numerical observations of^35^, which shows that these numerical observations are not only valid for a three-species cyclic competition system, but also apply to any *n*-species tournament for which a coexistence equilibrium exists.

**Notation**: Below is a list of symbols and notations used in this paper.

**Table:** 

Symbol	Description
${\mathbb {R}}_+^{n}$	The set of *n*-dimensional real vectors with strictly positive entries only
$x_i$	The *i*th element of a column vector ${\mathbf {x}}=(x_1,\ldots ,x_m)^\top \in {\mathbb {R}}^m$
$\mathbb {1}^m$	The vector of dimension *m* with all entries equal to 1
${\mathbf {0}}^m$	The vector of dimension *m* with all entries equal to 0
$S^{n}$	The unit simplex $\{{\mathbf {x}}\in {\mathbb {R}}^n \mid \sum _{i=1}^{n}x_{i}=1, 0\le x_{i}\le 1\}$ spanned by the standard unit vectors ${\mathbf {e}}_i$
$S_{+}^{n}$	The interior of $S^n$
$\frac{{\mathbf {x}}}{{\mathbf {z}}}$	The element-wise quotient vector with $\left( \frac{{\mathbf {x}}}{{\mathbf {z}}}\right) _i := \frac{x_i}{z_i}$
$\text {Ln } {\mathbf {x}}$	The element-wise logarithm with $(\text {Ln } {\mathbf {x}})_i:=\ln (x_i)$
$\text {Exp}({\mathbf {x}})$	The element-wise exponential, with $\left( \text {Exp}({\mathbf {x}})\right) _i:=e^{x_i}$
$A_{ij}$	The entry in the *i*th row and *j*th column of the matrix ${\mathbf {A}}$
${\mathbf {A}}^\top$	The transpose of matrix ${\mathbf {A}}$
$\text{ Im }({\mathbf {A}})$	The image (or range) of matrix ${\mathbf {A}}$
$\text{ diag }({\mathbf {x}})$	The diagonal matrix with as entries the elements of ${\mathbf {x}}$ (in the same order)

## Preliminaries

In this section, we highlight some terminologies, methods, and results from literature that will be used in this paper.

### Species interactions and tournament matrices

**Figure 1:**
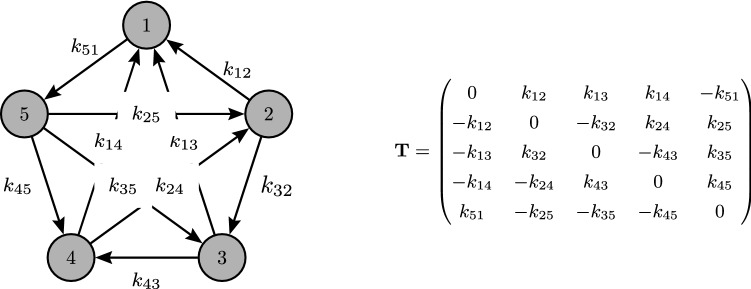
A 5-species tournament with corresponding tournament matrix.

We shall assume, like in many theoretical approaches, that, within each patch, each species can compete against every other species and that this competition is asymmetric such that for any pair of species, one is dominant over the other. These interactions can be represented by the paradigmatic reactions:

$$\begin{aligned} i+j \xrightarrow []{k_{ij}}i+i, \end{aligned}$$

where species *i* dominates species *j* at a rate $k_{ij}$. In this way, the interactions among species within a patch can be represented by a complete directed graph (or tournament) $G_1=(V_1,E_1)$ where $V_1=\{1,\ldots ,n\}$ is the set of species (vertices) and $E_1$ is the set of the edges (arrows) that point from the competitive subordinate to the competitive dominant. An edge from node *j* to node *i* in the graph $G_1$ is assigned a weight equal to $k_{ij}$. Equivalently, this tournament can be coded in an $n\times n$ tournament matrix ${\mathbf {T}}$ in which $T_{ij}=k_{ij}$ if species *i* outcompetes species *j* at a rate $k_{ij}$ and $T_{ij}=-k_{ji}$ if species *j* outcompetes species *i*. If $i=j$, then $T_{ij}$ is set to 0. We refer to Fig. 1 for an example.

#### Mean-field model and coexistence equilibrium

In a single patch system, we assume that the population dynamics are governed by a mean-field model that tracks the deterministic time evolution of the proportions of competing species assuming that: (1) there are a large number of competing individuals within the patch and (2) the contact rates between members of the competing species follow a mass-action rule. Thus, the intra-patch dynamics are described by a system of ordinary differential equations (ODE system):

1 $$\begin{aligned} {\dot{x}}_{i}=x_{i}\left( {\mathbf {T}}{\mathbf {x}}\right) _{i}, \end{aligned}$$

where $x_{i}\in [0,1]$ denotes the proportion of species *i* and ${\mathbf {x}}=\left( x_1,\ldots ,x_n\right) ^\intercal$. An equilibrium point (equilibrium from here on) ${\mathbf {x}}^*\in \, S^n$ of this system satisfies

$$\begin{aligned} x_{i}^*\left( {\mathbf {T}}{\mathbf {x}}^*\right) _i=0, \quad  i=1,\ldots ,n. \end{aligned}$$

An equilibrium ${\mathbf {x}}^*\in \,S_{+}^{n}$ is called a coexistence equilibrium. To guarantee the existence of such an equilibrium, the linear system of equations

$$\begin{aligned} {\mathbf {T}}{\mathbf {u}}={\mathbf {0}}^n \end{aligned}$$

must have a solution in ${\mathbb {R}}_+^n$. Obviously, if ${\mathbf {u}}$ is a solution, then also $q{\mathbf {u}}$ is, for any $q\in {\mathbb {R}}_+$. Hence, if there is a solution, then the mean-field model admits a coexistence equilibrium ${\mathbf {x}}^*\in S_{+}^{n}$.

#### Relationship with game theory

The properties (i.e., existence and stability) of the coexistence equilibrium of the mean-field ODE system (1) can be deduced from the behavior of the Nash equilibrium (NE) strategy of a corresponding zero-sum game. This is because the ODE system (1) is equivalent with the celebrated replicator equation of evolutionary game theory, which describes how a population of pure strategies evolves over time in a symmetric, two-player zero-sum game defined by the payoff matrix ${\mathbf {T}}$^10,39,40^. Here, the *n* species represent a set of pure strategies available to each player and $x_i$ represents the probability of a player using the pure strategy *i* at time *t*. A strategy for a player is a vector ${\mathbf {x}}=(x_1,\ldots ,x_n)^\top \in S^n$ indicating the probabilities of using the *n* pure strategies at time *t*. A strategy is pure if it is a unit vector, otherwise it is mixed. A strategy ${\mathbf {x}}$ is called completely mixed if it belongs to $S_{+}^{n}$. Solving an evolutionary game amounts to finding an optimal strategy ${\mathbf {x}}^*\in S^n$, called a Nash equilibrium (NE), such that for all ${\mathbf {y}}\in S^n$ it holds that

$$\begin{aligned} {\mathbf {y}}\cdot {\mathbf {T}}{\mathbf {x}}^*\le 0. \end{aligned}$$

The NE is said to be strict if equality holds only for ${\mathbf {y}}={\mathbf {x}}^*$.

The NE of a symmetric matrix game is closely related to the equilibrium of the replicator Eq. (1) via a theorem known as the *folk theorem of evolutionary game theory*^41^, which states that;

- ${\mathbf {x}}^*$ is an equilibrium of (1) if ${\mathbf {x}}^*\in S^n$ is a NE of the game defined by the payoff matrix ${\mathbf {T}}$; and
- ${\mathbf {x}}^*$ is a NE of the game defined by the payoff matrix ${\mathbf {T}}$ if ${\mathbf {x}}^*$ is a stable equilibrium of (1) or ${\mathbf {x}}^*$ is the $\omega$-limit of an orbit ${\mathbf {x}}(t)$ in $S^n_+$.

It has already been shown that every symmetric matrix game admits at least one NE^41^, so that System (1) is guaranteed to have at least one equilibrium. In particular, the vertices ${\mathbf {e}}_{i}$ of the simplex $S^n$ are always equilibria of (1). In addition, the simplex $S^n$ contains one or no equilibrium in its interior^39^. Thus, if a coexistence equilibrium for System (1) exists, then it is unique.

Since $\sum _{i=1}^{n}{\dot{x}}_{i}=0$, any solution of System (1) that starts on the plane $\sum _{i=1}^{n}x_{i}=1$ remains there. Furthermore, if $x_i(0)=0$, then $x_i(t)=0$ for all *t*, so that the faces of the simplex $S^n$, and therefore $S^n$ itself, are invariant^39^. The same holds for all sub-simplices of $S^n$ (which are given by $x_i=0$ for one or several *i*).

In this work, we are interested in System (1) for which a coexistence equilibrium exists and is stable. Thus, we consider competition networks for which the corresponding matrix game is completely mixed. In other words, games in which the optimal strategy NE is unique and completely mixed. It has already been shown that all two-person zero-sum games with skew-symmetric payoff matrices of even order are never completely mixed and hence the coexistence of an even number of species is not possible^42^. Kaplansky provided necessary and sufficient conditions for the game to be completely mixed^43^. That is, a two-player, symmetric, zero-sum game is completely mixed if its payoff matrix ${\mathbf {T}}$ has rank $n-1$ and all of its cofactors are different from zero and have the same sign. Furthermore, for games whose payoff matrices are of order 3 or 5, he went ahead to provide specific conditions for the game to be completely mixed. For instance, for $n=3$, the game with pay-off matrix

$$\begin{aligned} {\mathbf {T}}= \left[ \begin{array}{rrr} 0 &{} c &{} -b\\ -c &{} 0 &{} a\\ b &{} -a &{} 0 \end{array}\right] \end{aligned}$$

is completely mixed if and only if *a*, *b*, *c* are different from zero and have the same sign. The unique optimal strategy is then given by $(a/(a+b+c),~b/(a+b+c),~c/(a+b+c))$. For $n=5$, the symmetric game is completely mixed if and only if the following five expressions

$$\begin{aligned}&T_{25}T_{34}-T_{35}T_{24}+T_{45}T_{23}\\ -&T_{15}T_{34}+T_{35}T_{14}-T_{45}T_{13}\\&T_{15}T_{24}-T_{25}T_{14}+T_{45}T_{12}\\ -&T_{15}T_{23}+T_{25}T_{13}-T_{35}T_{12}\\&T_{14}T_{23}-T_{24}T_{13}+T_{34}T_{12} \end{aligned}$$

have the same sign and the unique optimal strategy is then proportional to them. These conditions were later extended to odd-ordered skew-symmetric payoff matrices^44^.

Finding the stable coexistence equilibrium of (1) is thus equivalent to finding the optimal strategy NE of the corresponding completely mixed matrix game. Interestingly, the problem of finding the NE for a two-person, zero-sum symmetric matrix game can be reduced to solving an appropriately defined linear programming problem^45^.

#### Neutral stability

Following^46^, we show that if System (1) admits a coexistence equilibrium, then it is neutrally stable. In other words, we show that if all the species coexist, then their proportions cycle neutrally around the coexistence equilibrium. To do so, we consider the Lyapunov function

2 $$\begin{aligned} V({\mathbf {x}})=-\sum _{i=1}^{n}x_{i}^{*}\ln \frac{x_i}{x_{i}^{*}}. \end{aligned}$$

By Gibbs inequality^47^, $V({\mathbf {x}})$ is positive in $S_{+}^{n}$ and $V({\mathbf {x}})=0$ only if ${\mathbf {x}}={\mathbf {x}}^*$. Taking the time derivative of *V*, we have (using $T_{ij}=-T_{ji}$):

$$\begin{aligned} {\dot{V}}({\mathbf {x}})=-\sum _{i=1}^{n}x_{i}^{*}\frac{{\dot{x}}_{i}}{x_{i}}=-\sum _{i=1}^{n}x_{i}^{*}\left( {\mathbf {T}}{\mathbf {x}}\right) _{i}=-\sum _{i=1}^{n}x_{i}^{*}\left( \sum _{j=1}^{n}T_{ij}x_{j}\right) =\sum _{j=1}^{n}x_{j}\left( \sum _{i=1}^{n}T_{ji}x_{i}^{*}\right) =\sum _{j=1}^{n}x_{j}\left( {\mathbf {T}}{\mathbf {x}}^*\right) _{j}=0. \end{aligned}$$

Hence, *V* is a constant of motion: all orbits $t\rightarrow {\mathbf {x}}(t)$ of the mean-field model remain on constant level sets of *V*. This implies that all orbits in $S_{+}^{n}$ are closed orbits surrounding ${\mathbf {x}}^*$.

### Detailed-balanced single species mass action reaction networks

We first recall the concept of detailed-balancing from CRNT as it will be instrumental when deriving the main results of this paper. With this formulation, the modelling of dispersion among patches is carried out analogously as in the case of detailed-balanced mass action chemical reaction networks^37,38^. We briefly explain the relevant aspects.

Consider a network of *r* reversible chemical reactions occurring among the chemical species $C_1,C_2,\ldots ,C_m$. Each of these *r* reversible reactions has a species $C_j$ as substrate and another species $C_k$ as product (with $j\ne k$). Let $A_{jk}$ (resp. $A_{kj}$) denote the mass action rate constant of the forward (resp. reverse) reaction in (3):

3 $$\begin{aligned} C_j\rightleftharpoons C_k. \end{aligned}$$

Since all reactions are reversible, it holds that $A_{jk}>0 \Leftrightarrow A_{kj}>0$ and $A_{jk}=0 \Leftrightarrow A_{kj}=0$, for any $j\ne k$.

We associate a finite asymmetric directed graph $G_2=(V_2,E_2)$ with the reaction network, where $V_2=\{1,\ldots ,m\}$ is the set of species and an edge (*j*, *k*) in $E_2$ is associated with each reversible reaction (3). The *incidence matrix* ${\mathbf {B}}\in {\mathbb {R}}^{m\times r}$ associated with the graph $G_2$ (see, e.g.,^48^) is defined as follows:

$$\begin{aligned} B_{jp}={\left\{ \begin{array}{ll} \begin{array}{cl} -1, &{}\quad \text {if } C_j \text { is the substrate of the } p{{\text {th}}} \text{ reversible } \text{ reaction }\\ 1, &{}\quad \text {if } C_j \text { is the product of the } p{ {\text {th}}}\text { reversible reaction}\\ 0, &{}\quad \text {otherwise} \end{array}. \end{array}\right. } \end{aligned}$$

Let $x_j$ denote the concentration of species $C_j$, for $j=1,\ldots ,m$. Let $v_p$ denote the overall rate of the $p{ {\text {th}}}$ reaction in the direction of the $p{ {\text {th}}}$ edge in $G_2$. Then the dynamics of the chemical reaction network can be described by the equation

4 $$\begin{aligned} \dot{{\mathbf {x}}}={\mathbf {B}}{\mathbf {v}}, \end{aligned}$$

where ${\mathbf {x}}=(x_1, x_2, \ldots , x_m)^{\top }$ and ${\mathbf {v}}=(v_1, v_2, \ldots , v_r)^{\top }$. Since each reaction of the network is governed by mass action kinetics, if the $p{ {\text {th}}}$ reaction of the network is described by (3), then

5 $$\begin{aligned} v_p = A_{jk}x_j-A_{kj}x_k. \end{aligned}$$

Define $k_p^{\text {forw}}:=A_{jk}$ and $k_p^{\text {rev}}:=A_{kj}$. A thermodynamic equilibrium for the network is a vector of equilibrium concentrations ${\mathbf {x}}^{*}\in {\mathbb {R}}_{+}^m$ for which ${\mathbf {v}}={\mathbf {0}}^r$. A reversible chemical reaction network is said to be *detailed balanced* if it admits a thermodynamic equilibrium. In other words, a detailed-balanced single species reversible reaction network is one for which there exists an equilibrium ${\mathbf {x}}^*$ at which the overall reaction rate of every reversible reaction of the network is zero. Thus, if $x_j^*$ and $x_k^*$ denote the concentrations of $C_j$ and $C_k$ at a thermodynamic equilibrium ${\mathbf {x}}^*$, then it holds that $A_{jk}x_j^*=A_{kj}{x_k^*}$. It is clear from Eq. (5) that if ${\mathbf {u}}$ is a thermodynamic equilibrium, then also $q{\mathbf {u}}$ is, for any $q\in {\mathbb {R}}_+$. Hence, we can choose a thermodynamic equilibrium ${\mathbf {z}}^*\in S_+^m$. Now define

$$\begin{aligned} K_p^{\text {eq}}:=\frac{z_k^*}{z_j^*}=\frac{A_{jk}}{A_{kj}}=\frac{k_p^{\text {forw}}}{k_p^{\text {rev}}}. \end{aligned}$$

Define ${\mathbf {K}}^{\text {eq}}:=(K_1^{\text {eq}},K_2^{\text {eq}},\ldots ,K_r^{\text {eq}})^{\top }$ and note that ${\mathbf {K}}^{\text {eq}}=\text {Exp}({\mathbf {B}}^{\top }\text {Ln}({\mathbf {z}}^*))$. From this, the condition for detailed balancing of a reversible single species chemical reaction network can be derived.

#### **Proposition 1**

*A reversible single species mass action chemical reaction network is detailed balanced if and only if*

6 $$\begin{aligned} {\text {Ln}}\left( {\mathbf {K}}^{\text {eq}}\right) \in \mathrm {\text {Im}}\big ({\mathbf {B}}^{\top }\big ). \end{aligned}$$

As mentioned in (^37^, Remark 3.1), from condition (6), it follows that any ${\mathbf {w}} \in {\mathbb {R}}^r$ satisfying ${\mathbf {Bw}}={\mathbf {0}}^m$ will also satisfy $\sum _{p=1}^rw_p\ln \left( K_p^{\text {eq}}\right) =0$. This leads to the well-known Wegscheider conditions^49^ for detailed balancing given by

$$\begin{aligned} \prod _{p=1}^r\left( k_p^{\text {forw}}\right) ^{w_p}=\prod _{p=1}^r\left( k_p^{\text {rev}}\right) ^{w_p}. \end{aligned}$$

Hence, the reversible reaction network

7 $$\begin{aligned} C_2 {\rightleftharpoons } C_1 {\rightleftharpoons } C_3 \end{aligned}$$

with strictly positive rate constants is always detailed balanced, whereas the cyclic reversible reaction networkwith strictly positive rate constants is detailed balanced if and only if $A_{12}A_{23}A_{31}=A_{21}A_{32}A_{13}$.

We now describe the compact mathematical formulation for a detailed-balanced network derived in^37^. This formulation will be crucially used for deriving the main results of this paper. Let (3) denote the $p{ {\text {th}}}$ reaction of a detailed-balanced single species network with a thermodynamic equilibrium ${\mathbf {z}}^*\in S^m_+$. Define

$$\begin{aligned} \kappa _p:=A_{jk}z_j^*=A_{kj}z_k^*. \end{aligned}$$

For any other vector of concentrations ${\mathbf {x}}\in {\mathbb {R}}_{+}^m$, it follows from Eq. (5) that the overall rate of the $p{ {\text {th}}}$ reaction in the forward direction is given by

$$\begin{aligned} v_p=\kappa _p\left( \frac{x_j}{z_j^*}-\frac{x_k}{z_k^*}\right) . \end{aligned}$$

Define ${\mathcal {K}}:= \text { diag}(\kappa _1,\kappa _2,\ldots ,\kappa _r)$. Then it can be verified that the vector ${\mathbf {v}}$ of reaction rates is given by

$$\begin{aligned} {\mathbf {v}}=-{\mathcal {K}}{\mathbf {B}}^{\top }\left( \frac{{\mathbf {x}}}{{\mathbf {z}}^*}\right) . \end{aligned}$$

Define ${\mathbf {Z}}^*:=\text { diag}({\mathbf {z}}^*)$. From Eq. (4), it now follows that the dynamics of the detailed-balanced single species reaction network is described by the equation

8 $$\begin{aligned} \dot{{\mathbf {x}}}=-{\mathbf {B}}{\mathcal {K}}{\mathbf {B}}^{\top }\left( \frac{{\mathbf {x}}}{{\mathbf {z}}^*}\right) =-\left( {\mathbf {B}}{\mathcal {K}}{\mathbf {B}}^{\top }({\mathbf {Z}}^*)^{-1}\right) {\mathbf {x}}. \end{aligned}$$

Equation (8) will be used to provide an analogous formulation for the modelling of migrations of species among the different habitat patches of the metapopulation.

## Metapopulation models

We now derive the metapopulation model used in this paper. We start by deriving a general metapopulation model that is based on the seminal work of Levin^50^. Assuming that the inter-patch migrations are detailed-balanced, we make use of the formulation in Eq. (8) to derive a balanced metapopulation model. We then show that the balanced model admits a unique coexistence equilibrium that is asymptotically stable if the dispersal network is heterogeneous, whereas the same equilibrium is neutrally stable in the case of a homogeneous network.

### General metapopulation model

Mathematical models based on traditional metapopulation theory usually assume that the metapopulation is made up of many neighboring spatially homogeneous habitat patches connected via dispersal. Consider an interconnected network of *m* discrete patches each being inhabited by the same *n* species. In addition, assume that species can migrate from one patch to some or all of the other patches. The rate of migration of each species between two patches is directly proportional to the proportion of the particular species in the originating patch, with a (nonnegative) constant of proportionality being the same across species. This constant of proportionality will be referred to as the rate constant associated with the migration. It is assumed that if there is migration between two given patches, then it is bidirectional, i.e., the rate constant of migration from *j* to *k* is strictly positive if and only the same holds for the migration from *k* to *j*. Just like in the case of a reversible single-species chemical reaction network, inter-patch migrations may be described by a weighted symmetric directed graph $G_2=(V_2,E_2)$ where $V_2=\{1,\ldots ,m\}$ is the set of patches (vertices) and an edge $(j,k)\in E_2$ means that every species can migrate from patch *j* to patch *k*. Finally, it is also assumed that the graph $G_2$ corresponding to the inter-patch migration is *connected*, i.e., there is a path between every two distinct vertices of the graph.

The flow of species between the patches can be summarized in a weighted $m\times m$ adjacency matrix ${\mathbf {A}}$ with entry $A_{jk}$ being equal to the rate constant of migration of species from the $j{ {\text {th}}}$ to the $k{ {\text {th}}}$ patch. The diagonal elements of ${\mathbf {A}}$ are hence equal to 0. Due to the bidirectional nature of migration, it holds that $A_{jk}>0 \Leftrightarrow A_{kj}>0$ and $A_{jk}=0 \Leftrightarrow A_{kj}=0$, for any $j\ne k$. Let $\Delta =\text {diag}(\delta _1,\ldots ,\delta _m)$ denote the *m*-dimensional diagonal matrix whose $j{ {\text {th}}}$ entry is given by

$$\begin{aligned} \delta _{j}=\sum _{k=1}^{m} A_{jk}. \end{aligned}$$

Define ${\mathbf {L}}:=\Delta -{\mathbf {A}}^\top$. Note that

$$\begin{aligned} (\mathbb {1}^m)^{\top }{\mathbf {L}}=(\mathbb {1}^m)^{\top }\Delta -\big ({\mathbf {A}}\mathbb {1}^m\big )^{\top }=({\mathbf {0}}^m)^\top . \end{aligned}$$

Let ${\mathbf {x}}\in S^{mn}$, with $x_{i,j}$ the proportion of species *i* in patch *j* across the entire metapopulation, then the net migration rate $\psi _{i,j}$ of species *i* from other patches to patch *j* is given by

$$\begin{aligned} \psi _{i,j}=\sum _{k=1}^{m}A_{kj}x_{i,k}-\sum _{k=1}^{m}A_{jk}x_{i,j}=\sum _{k=1}^{m}A_{kj}x_{i,k}-\delta _{j}x_{i,j}=-\sum _{k=1}^{m}L_{jk}x_{i,k}. \end{aligned}$$

Let us denote $\Psi _i:=\left( \psi _{i,1},\psi _{i,2},\ldots ,\psi _{i,m}\right) ^{\top }$ and ${\mathbf {r}}_i:=\left( x_{i,1},x_{i,2},\ldots ,x_{i,m}\right) ^{\top }$, then

9 $$\begin{aligned} \Psi _{i}=-{\mathbf {L}}{\mathbf {r}}_{i}. \end{aligned}$$

Within each patch, the proportions of species are affected by other patches only via migration. Let $\phi _{i,j}$ denote the rate of change of the proportion of species *i* in patch *j* in the absence of migration. Since the dominance relationships among the species (described by a tournament matrix ${\mathbf {T}}$) are assumed to be the same for all patches and since the habitat patches are spatially homogeneous, the expression for $\phi _{i,j}$ is given by the right-hand side of System (1):

10 $$\begin{aligned} \phi _{i,j}=x_{i,j}\left( {\mathbf {T}}{\mathbf {p}}_{j}\right) _{i}, \end{aligned}$$

where ${\mathbf {p}}_j:=\left( x_{1,j},x_{2,j},\ldots , x_{n,j}\right) ^{\top }$, $i=1,\ldots ,n$ and $j=1,\ldots ,m$. Assuming migration among the patches, the proportion of a species within a patch is influenced by two factors: the first is the interaction with other species within the patch and the second is the migration of that particular species to or from other patches. Thus, the metapopulation model describing the dynamics of the *n* species in the *m*-patch network is described by the system of *mn* differential equations;

11 $$\begin{aligned} {\dot{x}}_{i,j}=\phi _{i,j}+\psi _{i,j}=x_{i,j}\left( {\mathbf {T}}{\mathbf {p}}_{j}\right) _{i}-\left( {\mathbf {L}}{\mathbf {r}}_{i}\right) _{j},\qquad i=1,\ldots ,n,\quad j=1,\ldots ,m . \end{aligned}$$

This system evolves on the unit simplex $S^{mn}$.

#### **Proposition 2**

*The unit simplex* $S^{mn}$ *is positively invariant for System* (11).

#### Proof

To show the invariance of the unit simplex $S^{mn}$ under the flow of System (11), it suffices to show that each of the faces of the simplex cannot be crossed, i.e., the vector field points inward from the faces of $S^{mn}$.

On the one hand, if $x_{i,j}=0$ for some *i*, *j*, then

$$\begin{aligned}{\dot{x}}_{i,j}=\sum _{k=1}^{m}A_{kj}x_{i,k}\ge 0,\end{aligned}$$

which implies that $x_{i,j}=0$ cannot be crossed from positive to negative. In an ecological context, this condition simply states the obvious fact that an extinct species is in no danger of declining. On the other hand, if $x_{i,j}=1$ for some *i*, *j*, then obviously $x_{l,k}=0$ for any $l\ne i$ or $k\ne j$ and

$$\begin{aligned}{\dot{x}}_{i,j}=-\delta _{j}< 0.\end{aligned}$$

Hence, the vector field associated with System (11) points inward from the faces of $S^{mn}$. So, $S^{mn}$ is positively invariant under the flow of System (11). $\square$

Note that Proposition 2 does not exclude the solution trajectories of System (11) from approaching the boundary equilibria of the system as $t\rightarrow \infty$. We call metapopulation model (11) *persistent* if for every ${\mathbf {x}}_0\in S^{mn}_{+}$, the $\omega$-limit set $\omega ({\mathbf {x}}_0)$ does not intersect the boundary of $S^{mn}$. In other words, a metapopulation model is persistent if the initial existence of all the species implies that none of the species goes extinct with the passage of time.

### Balanced homogeneous and heterogeneous metapopulation models

We say that the inter-patch migration of a metapopulation model is *detailed balanced* if the overall migration rate of any species between any two patches is zero for a certain positive set of proportions of that species in the different patches. From the theory of detailed-balanced reaction networks described in “Detailed-balanced single species mass action reaction networks” section, it follows that a detailed-balanced inter-patch migration network corresponds to a detailed-balanced single species mass action reaction network. Let *B* denote the incidence matrix corresponding to the directed graph $G_2$ describing the inter-patch migrations and let *r* denote the number of edges in $G_2$. Comparing Eqs. (8) and (9), it follows that if the inter-patch migration is detailed balanced, then there exist diagonal matrices ${\mathcal {K}}\in {\mathbb {R}}^{r\times r}$ and ${\mathbf {Z}}^*\in {\mathbb {R}}^{m\times m}$ with positive diagonal entries such that $(\mathbb {1}^m)^{\top }{\mathbf {Z}}^*\mathbb {1}^m=1$ and

$$\begin{aligned} {\mathbf {L}}={\mathbf {B}}{\mathcal {K}}{\mathbf {B}}^{\top }({\mathbf {Z}}^*)^{-1}. \end{aligned}$$

Let ${\mathbf {Z}}^*=\text { diag}({\mathbf {z}}^*)$. Equation (9) can now be rewritten as

12 $$\begin{aligned} \Psi _{i}=-{\mathbf {B}}{\mathcal {K}}{\mathbf {B}}^{\top }\left( \frac{{\mathbf {r}}_{i}}{{\mathbf {z}}^{*}}\right) . \end{aligned}$$

Henceforth in this manuscript, we restrict our analysis to metapopulation models of type (11) for which the interactions within each patch correspond to a tournament with a completely mixed optimal strategy and whose inter-patch migration is detailed balanced. Such metapopulation models will be referred to as *balanced metapopulation models*.

We have seen earlier in “Species interactions and tournament matrices” section that if the interactions within every patch correspond to a tournament with a completely mixed optimal strategy, then the corresponding mean-field model admits a unique coexistence equilibrium ${\mathbf {y}}^*\in S^{n}_{+}$ with ${\mathbf {T}}{\mathbf {y}}^*={\mathbf {0}}^n$. Thus, for a balanced metapopulation model, System (10) can be rewritten as

13 $$\begin{aligned} \phi _{i,j}=x_{i,j}\left( \mathbf {TY}^*\left( \frac{{\mathbf {p}}_{j}}{{\mathbf {y}}^*}\right) \right) _i, \end{aligned}$$

where ${\mathbf {Y}}^*:=$ diag$({\mathbf {y}}^*)$. Consequently, from Eqs. (11)–(13), it follows that the dynamics of a balanced metapopulation model containing *n* species and *m* patches can be described by *mn* differential equations

14 $$\begin{aligned} {\dot{x}}_{i,j}=x_{i,j}\left( \mathbf {TY}^*\left( \frac{{\mathbf {p}}_{j}}{{\mathbf {y}}^*}\right) \right) _i-\left( {\mathbf {B}}{\mathcal {K}}{\mathbf {B}}^{\top }\left( \frac{{\mathbf {r}}_{i}}{{\mathbf {z}}^{*}}\right) \right) _{j} ,\qquad i=1,\ldots ,n,\quad j=1,\ldots ,m . \end{aligned}$$

If all the elements of ${\mathbf {z}}^*$ in the above equation are equal, i.e., if $z_j^*=\frac{1}{m}$ for $j=1,\ldots ,m$, then we say that the balanced metapopulation model is *homogeneous*, otherwise we call it *heterogeneous*. Whether a balanced metapopulation model is homogeneous or not can be checked from the adjacency matrix ${\mathbf {A}}$ corresponding to its inter-patch migration graph $G_2$. If ${\mathbf {A}}$ is symmetric, then the model is homogeneous, otherwise it is heterogeneous.

#### *Remark 3*

**Figure 2:**
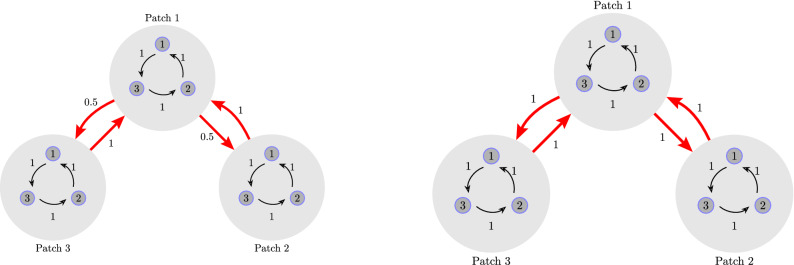
Left: A heterogeneous dispersal graph according to^35^. Right: A homogeneous dispersal graph according to our definition.

In^35^, the authors assume that migrations from one patch to other patches are random with a probability of migration (or migration constant) equal to the reciprocal of the number of dispersal links from a patch to other patches. They thus define a dispersal graph to be homogeneous if all nodes have the same degree (number of links), otherwise the graph is heterogeneous. With this definition, homogeneity, in general, is equivalent to the existence of cycles in the dispersal graph, whereas heterogeneity is equivalent to their absence. However, with our new definition, it is clear that this is not necessary. An example of such a case is shown in Fig. 2.

### Coexistence equilibrium and its uniqueness

In this section, we present a theorem that gives an expression for a coexistence equilibrium of a balanced metapopulation model. Before we state our main theorem in this section, we need the following lemma.

#### **Lemma 4**

*Let* ${\mathbf {B}}\in {\mathbb {R}}^{m\times r}$ *denote the incidence matrix of a finite connected directed graph* $G_2$ *and let* ${\mathcal {K}}\in {\mathbb {R}}^{r\times r}$ *denote a diagonal matrix with positive diagonal entries*. *For any* ${\mathbf {w}}\in {\mathbb {R}}_+^m$, *it holds that* $-{\mathbf {w}}^{\top }{\mathbf {B}}{\mathcal {K}}{\mathbf {B}}^{\top }\left( \frac{\mathbb {1}^m}{{\mathbf {w}}}\right) \ge 0$. *Moreover* $-{\mathbf {w}}^{\top }{\mathbf {B}}{\mathcal {K}}{\mathbf {B}}^{\top }\left( \frac{\mathbb {1}^m}{{\mathbf {w}}}\right) = 0$ *if and only if* ${\mathbf {w}}=q\mathbb {1}^m$, *where* $q\in {\mathbb {R}}_+$.

#### Proof

Assume that the $p{ {\text {th}}}$ edge of the graph $G_2$ is directed from vertex $i_p$ to vertex $j_p$. Hence, $B_{i_pp}=-1$, $B_{j_pp}=1$ and $B_{kp}=0$ for $i_p\ne k\ne j_p$. Thus,

$$\begin{aligned} -{\mathbf {w}}^{\top }{\mathbf {B}}{\mathcal {K}}{\mathbf {B}}^{\top }\left( \frac{\mathbb {1}^m}{{\mathbf {w}}}\right) =\sum _{p=1}^m(w_{j_p}-w_{i_p})\kappa _p\left( \frac{1}{w_{i_p}}-\frac{1}{w_{j_p}}\right) =\sum _{p=1}^m\frac{\kappa _p}{w_{i_p}w_{j_p}}\left( w_{j_p}-w_{i_p}\right) ^2\ge 0. \end{aligned}$$

Moreover, $-{\mathbf {w}}^{\top }{\mathbf {B}}{\mathcal {K}}{\mathbf {B}}^{\top }\left( \frac{\mathbb {1}^m}{{\mathbf {w}}}\right) =0$ if and only if $w_{j_p}=w_{i_p}$ for $p=1,\ldots ,m$, which is equivalent with ${\mathbf {B}}^{\top }{\mathbf {w}}={\mathbf {0}}^r$.

Since the graph $G_2$ is connected, we recall from^48^ that $\text {rank}({\mathbf {B}})=m-1$ and furthermore $\text {ker}({\mathbf {B}}^{\top })=\mathbb {1}^m$. Therefore ${\mathbf {B}}^{\top }{\mathbf {w}}={\mathbf {0}}^r$ if and only if ${\mathbf {w}}=q\mathbb {1}^m$, where $q\in {\mathbb {R}}_+$. This completes the proof. $\square$

We now state the main theorem of this section.

#### **Theorem 5**

*A balanced metapopulation model described by System* (14) *admits a unique coexistence equilibrium* ${\mathbf {x}}^*\in S^{mn}_{+}$. *The proportion* $x_{i,j}^{*}$ *of species i in patch j at the unique coexistence equilibrium is given by*

15 $$\begin{aligned} x_{i,j}^*=y^{*}_iz^{*}_j. \end{aligned}$$

*for* $$i=1,\ldots ,n$$ *and* $$j=1,\ldots ,m$$.

#### Proof

We divide the proof into two parts. In the first part we prove that System (15) indeed yields an equilibrium for the model. In the second part, we prove that this coexistence equilibrium is unique.

Let us define

$$\begin{aligned} {\mathbf {p}}_{j}^*:=\left( x_{1,j}^*, x_{2,j}^*, \ldots , x_{n,j}^*\right) ^\top =z_j^*{\mathbf {y}}^*; \quad  {\mathbf {r}}_{i}^{*}:=\left( x_{i,1}^{*}, x_{i,2}^{*}, \ldots , x_{i,m}^{*}\right) ^\top =y_i^*{\mathbf {z}}^*. \end{aligned}$$

For ${\mathbf {x}}^*$ to be an equilibrium of System (14), it should render the right-hand side equal to zero. Note that

$$\begin{aligned} \mathbf {TY}^*\left( \frac{{\mathbf {p}}_{j}^*}{{\mathbf {y}}^*}\right) =z_j^*\mathbf {TY}^*\mathbb {1}^n=z_j^*{\mathbf {T}}{\mathbf {y}}^{*}={\mathbf {0}}^n \end{aligned}$$

and

$$\begin{aligned} {\mathbf {B}}{\mathcal {K}}{\mathbf {B}}^{\top }\left( \frac{{\mathbf {r}}_{i}^*}{{\mathbf {z}}^{*}}\right) =y_i^*{\mathbf {B}}{\mathcal {K}}{\mathbf {B}}^{\top }\mathbb {1}^m={\mathbf {0}}^m. \end{aligned}$$

In addition,

$$\begin{aligned} (\mathbb {1}^{mn})^{\top }{\mathbf {x}}^*=\sum _{i=1}^n\sum _{j=1}^mx_{i,j}^*=\sum _{i=1}^{n}y_i^*\sum _{j=1}^mz_j^{*}=1. \end{aligned}$$

Thus, ${\mathbf {x}}^*$ is a coexistence equilibrium of System (14).

Assume that there exists another coexistence equilibrium $${\mathbf {x}}^{**}\in \, S^{mn}_{+}$$. Let $$x_{i,j}^{**}$$ denote the corresponding proportion of species *i* in patch *j* and define

$$\begin{aligned} {\mathbf {p}}_{j}^{**}:=\left( x_{1,j}^{**}, x_{2,j}^{**}, \ldots , x_{n,j}^{**}\right) ^\top ; \qquad {\mathbf {r}}_{i}^{**}:=\left( x_{i,1}^{**}, x_{i,2}^{**}, \ldots , x_{i,m}^{**}\right) ^\top . \end{aligned}$$

It follows that for any *i*, *j* it holds that

16 $$\begin{aligned} x_{i,j}^{**}\left( \mathbf {TY}^*\left( \frac{{\mathbf {p}}_{j}^{**}}{{\mathbf {y}}^*}\right) \right) _i-\left( {\mathbf {B}}{\mathcal {K}}{\mathbf {B}}^{\top }\left( \frac{{\mathbf {r}}_{i}^{**}}{{\mathbf {z}}^{*}}\right) \right) _{j}=0. \end{aligned}$$

Multiplying both sides of this equality with $$\frac{x_{i,j}^*}{x_{i,j}^{**}}$$, we get

$$\begin{aligned} x_{i,j}^*\left( \mathbf {TY}^*\left( \frac{{\mathbf {p}}_{j}^{**}}{{\mathbf {y}}^*}\right) \right) _i- \frac{x_{i,j}^*}{x_{i,j}^{**}} \left( {\mathbf {B}}{\mathcal {K}}{\mathbf {B}}^{\top }\left( \frac{{\mathbf {r}}_{i}^{**}}{{\mathbf {z}}^{*}}\right) \right) _{j}=0. \end{aligned}$$

Summing the left-hand side of the above expression over the different species and patches, we get

17 $$\begin{aligned} \sum _{j=1}^m\sum _{i=1}^nx_{i,j}^*\left( \mathbf {TY}^*\left( \frac{{\mathbf {p}}_{j}^{**}}{{\mathbf {y}}^*}\right) \right) _i- \sum _{i=1}^n\sum _{j=1}^m\frac{x_{i,j}^*}{x_{i,j}^{**}} \left( {\mathbf {B}}{\mathcal {K}}{\mathbf {B}}^{\top }\left( \frac{{\mathbf {r}}_{i}^{**}}{{\mathbf {z}}^{*}}\right) \right) _{j}=0. \end{aligned}$$

Now consider the two terms in the left-hand side of the above equality separately. For the first term, note that for any *j* it holds that

$$\begin{aligned} \sum _{i=1}^nx_{i,j}^*\left( \mathbf {TY}^*\left( \frac{{\mathbf {p}}_{j}^{**}}{{\mathbf {y}}^*}\right) \right) _i= & {} \sum _{i=1}^nx_{i,j}^*\left( {\mathbf {T}}{\mathbf {p}}_{j}^{**}\right) _{i} = \sum _{i=1}^{n}x_{i,j}^{*}\left( \sum _{l=1}^{n}T_{il}x_{l,j}^{**}\right) =-\sum _{l=1}^{n}x_{l,j}^{**}\left( \sum _{i=1}^{n}T_{li}x_{i,j}^{*}\right) \\= & {} -\sum _{l=1}^{n}x_{l,j}^{**}\left( \sum _{i=1}^nT_{li}y_i^{*}z_j^*\right) =-z_j^*\sum _{l=1}^{n}x_{l,j}^{**}({\mathbf {T}}{\mathbf {y}}^*)_l=0. \end{aligned}$$

Hence,

$$\begin{aligned} \sum _{j=1}^m\sum _{i=1}^nx_{i,j}^*\left( \mathbf {TY}^*\left( \frac{{\mathbf {p}}_{j}^{**}}{{\mathbf {y}}^*}\right) \right) _i=0. \end{aligned}$$

For the second term, we find

$$\begin{aligned} -\sum _{i=1}^n\sum _{j=1}^m\frac{x_{i,j}^*}{x_{i,j}^{**}} \left( {\mathbf {B}}{\mathcal {K}}{\mathbf {B}}^{\top }\left( \frac{{\mathbf {r}}_{i}^{**}}{{\mathbf {z}}^{*}}\right) \right) _{j}=-\sum _{i=1}^ny_i^*\sum _{j=1}^m\frac{z_j^*}{x_{i,j}^{**}}\left( {\mathbf {B}}{\mathcal {K}}{\mathbf {B}}^{\top }\left( \frac{{\mathbf {r}}_{i}^{**}}{{\mathbf {z}}^{*}}\right) \right) _{j}=-\sum _{i=1}^ny_i^*\left( \frac{{\mathbf {z}}^{*}}{{\mathbf {r}}_{i}^{**}}\right) ^{\top }{\mathbf {B}}{\mathcal {K}}{\mathbf {B}}^{\top }\left( \frac{{\mathbf {r}}_{i}^{**}}{{\mathbf {z}}^{*}}\right) . \end{aligned}$$

Thus, Eq. (17) can be simplified as

$$\begin{aligned} -\sum _{i=1}^ny_i^*\left( \frac{{\mathbf {z}}^{*}}{{\mathbf {r}}_{i}^{**}}\right) ^{\top }{\mathbf {B}}{\mathcal {K}}{\mathbf {B}}^{\top }\left( \frac{{\mathbf {r}}_{i}^{**}}{{\mathbf {z}}^{*}}\right) =0. \end{aligned}$$

Since $y_i^*>0$ for $i=1,\ldots ,n$, it holds for any $i=1,\ldots ,n$ that

18 $$\begin{aligned} -\left( \frac{{\mathbf {z}}^{*}}{{\mathbf {r}}_{i}^{**}}\right) ^{\top }{\mathbf {B}}{\mathcal {K}}{\mathbf {B}}^{\top }\left( \frac{{\mathbf {r}}_{i}^{**}}{{\mathbf {z}}^{*}}\right) =0. \end{aligned}$$

From Eq. (18) and Lemma 4, it follows that $${\mathbf {r}}_{i}^{**}=q_i{\mathbf {z}}^*$$ with $$q_i\in {\mathbb {R}}_+$$ for $$i=1,\ldots ,n$$. Thus, $$x_{i,j}^{**}=q_iz_{j}^*$$ and $${\mathbf {p}}_{j}^{**}=z_j^*{\mathbf {q}}$$ for $$i=1,\ldots ,n$$ and $$j=1,\ldots ,m$$. Substituting the latter in the left-hand side of Eq. (16), we get

$$\begin{aligned} x_{i,j}^{**}\left( \mathbf {TY}^*\left( \frac{{\mathbf {p}}_{j}^{**}}{{\mathbf {y}}^*}\right) \right) _i-\left( {\mathbf {B}}{\mathcal {K}}{\mathbf {B}}^{\top }\left( \frac{{\mathbf {r}}_{i}^{**}}{{\mathbf {z}}^{*}}\right) \right) _{j}=q_i{z_j^*}^2\left( {\mathbf {T}}{\mathbf {Y}}^*\left( \frac{{\mathbf {q}}}{{\mathbf {y}}^*}\right) \right) _i-q_i\left( {\mathbf {B}}{\mathcal {K}}{\mathbf {B}}^{\top }\mathbb {1}^m\right) _j=q_i{z_j^*}^2(\mathbf {Tq})_i. \end{aligned}$$

Since $q_i>0$ for $i=1,\ldots ,n$, for Eq. (16) to hold, we should have $\mathbf {Tq}={\mathbf {0}}^n$. Also note that

$$\begin{aligned} (\mathbb {1}^{mn})^{\top }{\mathbf {x}}^{**}=\sum _{i=1}^n\sum _{j=1}^{m}x_{i,j}^{**}=\sum _{i=1}^nq_i\sum _{j=1}^mz_j^* =\sum _{i=1}^nq_i=1. \end{aligned}$$

Since the metapopulation model is balanced, it follows that $${\mathbf {q}}={\mathbf {y}}^*$$. Thus, $$x_{i,j}^{**}=y_i^*z_j^*=x_{i,j}^*$$ for $$i=1,\ldots ,n$$ and $$j=1,\ldots ,m$$. This proves the uniqueness of the coexistence equilibrium $${\mathbf {x}}^*$$. $$\square $$

We now give examples of two balanced metapopulation models.

#### *Example 1*

**Figure 3:**
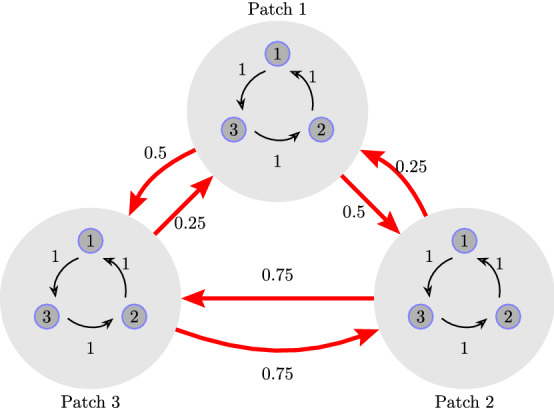
A metapopulation network composed of three patches. Each patch contains a local population composed of three species (1, 2 and 3), in cyclic competition, as shown by the black arrows. The red arrows denote migrations among the patches in the directions shown.

It is easy to verify that the network shown in Fig. 3 corresponds to a balanced metapopulation model governed by System (14) with

$$\begin{aligned} {\mathbf {T}} = \left[ \begin{array}{rrr} 0 &{}\quad 1 &{}\quad -1\\ -1 &{}\quad 0 &{}\quad 1\\ 1 &{}\quad -1 &{}\quad 0 \end{array}\right] ; \quad  {\mathbf {B}} = \left[ \begin{array}{rrr} -1 &{}\quad 0 &{}\quad 1\\ 1 &{}\quad -1 &{}\quad 0\\ 0 &{}\quad 1 &{}\quad -1 \end{array}\right] ; \end{aligned}$$

${\mathbf {y}}^*=\left( \frac{1}{3}, \frac{1}{3}, \frac{1}{3} \right) ^{\top }$, ${\mathbf {z}}^*=\left( \frac{1}{5}, \frac{2}{5}, \frac{2}{5} \right) ^{\top }$ and ${\mathcal {K}}=\text { diag}\left( \frac{1}{10},\frac{3}{10},\frac{1}{10}\right)$. Note that this metapopulation model is heterogeneous. From Theorem 5, it follows that the species proportions at the unique coexistence equilibrium for this model are given by $x_{i,1}^*=\frac{1}{15}$ and $x_{i,2}^*=x_{i,3}^*= \frac{2}{15}$.

#### *Example 2*

**Figure 4:**
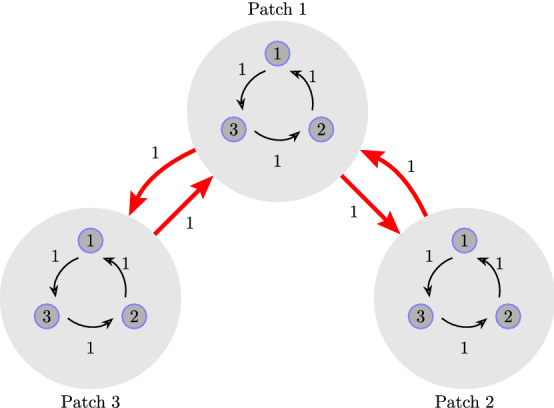
A metapopulation network composed of three patches. Species can migrate from patch 1 to the other two patches and vice versa. However, there exists no migrations between patches 2 and 3.

It is easy to verify that the network shown in Fig. 4 corresponds to a balanced metapopulation model governed by System (14) with

$$\begin{aligned} {\mathbf {T}} = \left[ \begin{array}{rrr} 0 &{}\quad 1 &{} \quad -1\\ -1 &{}\quad 0 &{} \quad 1\\ 1 &{}\quad -1 &{}\quad 0 \end{array}\right] ; \quad {\mathbf {B}} = \left[ \begin{array}{rrr} 1 &{}\quad -1\\ 0 &{}\quad 1\\ -1 &{}\quad 0 \end{array}\right] ; \end{aligned}$$

${\mathbf {y}}^{*}={\mathbf {z}}^*=\left( \frac{1}{3}, \frac{1}{3}, \frac{1}{3} \right) ^{\top }$ and ${\mathcal {K}}=\frac{1}{3}\text { diag}(\mathbb {1}_2)$. Note that this metapopulation model is homogeneous. From Theorem 5, it follows that the species proportions at the unique coexistence equilibrium in this case are all given by $x_{i,j}^*=\frac{1}{9}$ for $i,j= 1,2,3$.

### Stability

We now prove the local stability of the unique coexistence equilibrium corresponding to the balanced metapopulation model (14). For the proof, we make use of the same Lyapunov function as in “Neutral stability” section, coupled with LaSalle’s invariance principle^51^, (^52^, Section 4.2), (^53^, pp. 188–189).

#### **Theorem 6**

*Consider the balanced metapopulation model* (14) *with coexistence equilibrium* ${\mathbf {x}}^*\in \, S^{mn}_{+}$.

1. *If the model is heterogeneous, then* ${\mathbf {x}}^*$ *is locally asymptotically stable w.r.t. all initial conditions in* $S^{mn}_{+}$ *in the neighbourhood of* ${\mathbf {x}}^*$. *Furthermore, if the model is persistent, then* ${\mathbf {x}}^*$ *is globally asymptotically stable w.r.t. all initial conditions in* $S^{mn}_{+}$.
2. *If the model is homogeneous and persistent, then as* $t\rightarrow \infty$, *the solution trajectories converge to a limit cycle satisfying the equation* ${\dot{x}}_{i,j}=x_{i,j}({\mathbf {T}}{\mathbf {p}}_{j})_i$ *with* $x_{i,j}=x_{i,k}$, *for* $i=1,\ldots ,n$ *and* $j,k=1,\ldots ,m$.

#### Proof

Let $x_{i,j}$ denote the proportion of species *i* in patch *j*. Assuming that ${\mathbf {x}}\in S^{mn}_{+}$, consider the Lyapunov function

19 $$\begin{aligned} V({\mathbf {x}})=-(\mathbf {x^{*}})^{\top }\text {Ln}\left( \frac{{\mathbf {x}}}{{\mathbf {x}}^*}\right) . \end{aligned}$$

By Gibbs inequality, *V*(*x*) is positive on $S^{mn}_{+}$ and is equal to zero only if ${\mathbf {x}}={\mathbf {x}}^*$. Taking the time derivative of *V*, we have

$$\begin{aligned} {\dot{V}}({\mathbf {x}})=-\sum _{j=1}^m\sum _{i=1}^{n}\left( \frac{x_{i,j}^{*}}{x_{i,j}}\right) {\dot{x}}_{i,j}. \end{aligned}$$

From Eq. (14), it follows that

$$\begin{aligned} {\dot{V}}({\mathbf {x}})= -\sum _{j=1}^m\sum _{i=1}^nx_{i,j}^*\left( \mathbf {TY}^*\left( \frac{{\mathbf {p}}_{j}}{{\mathbf {y}}^*}\right) \right) _i+ \sum _{i=1}^n\sum _{j=1}^m\frac{x_{i,j}^*}{x_{i,j}} \left( {\mathbf {B}}{\mathcal {K}}{\mathbf {B}}^{\top }\left( \frac{{\mathbf {r}}_{i}}{{\mathbf {z}}^{*}}\right) \right) _{j}. \end{aligned}$$

As in the proof of Theorem 5, it can be verified that

$$\begin{aligned} \sum _{j=1}^m\sum _{i=1}^nx_{i,j}^*\left( \mathbf {TY}^*\left( \frac{{\mathbf {p}}_{j}}{{\mathbf {y}}^*}\right) \right) _i=0 \end{aligned}$$

and

$$\begin{aligned} \sum _{i=1}^n\sum _{j=1}^m\frac{x_{i,j}^*}{x_{i,j}} \left( {\mathbf {B}}{\mathcal {K}}{\mathbf {B}}^{\top }\left( \frac{{\mathbf {r}}_{i}}{{\mathbf {z}}^{*}}\right) \right) _{j}=\sum _{i=1}^ny_i^*\left( \frac{{\mathbf {z}}^{*}}{{\mathbf {r}}_{i}}\right) ^{\top }{\mathbf {B}}{\mathcal {K}}{\mathbf {B}}^{\top }\left( \frac{{\mathbf {r}}_{i}}{{\mathbf {z}}^{*}}\right) . \end{aligned}$$

Thus,

$$\begin{aligned} {\dot{V}}({\mathbf {x}})=\sum _{i=1}^ny_i^*\left( \frac{{\mathbf {z}}^{*}}{{\mathbf {r}}_{i}}\right) ^{\top }{\mathbf {B}}{\mathcal {K}}{\mathbf {B}}^{\top }\left( \frac{{\mathbf {r}}_{i}}{{\mathbf {z}}^{*}}\right) . \end{aligned}$$

Since $y_i^*>0$ for $i=1,\ldots ,n$, it follows from Lemma 4 that ${\dot{V}}({\mathbf {x}})\le 0$ and ${\dot{V}}({\mathbf {x}})=0$ if and only if ${\mathbf {r}}_i=q_i{\mathbf {z}}^*$ with $q_i\in {\mathbb {R}}_+$, for $i=1,\ldots ,n$. Thus,

20 $$\begin{aligned} x_{i,j}=q_iz_j^*, \end{aligned}$$

for $i= 1,\ldots ,n$ and $j=1,\ldots ,m$. Since $(\mathbb {1}^{mn})^{\top }{\mathbf {x}}=1$, we obtain

$$\begin{aligned} \sum _{i=1}^n\sum _{j=1}^{m}x_{i,j}=\sum _{i=1}^nq_i\sum _{j=1}^mz_j^* =\sum _{i=1}^nq_i=1. \end{aligned}$$

Let ${\mathcal {E}}\subset S^{mn}_{+}$ be the set of all vectors ${\mathbf {x}}$ for which condition (20) is satisfied with $(\mathbb {1}^n)^{\top }{\mathbf {q}}=1$. We now determine the largest subset of ${\mathcal {E}}$ that is positively invariant w.r.t. System (14). Assume that ${\mathbf {x}}$ continuously takes values from ${\mathcal {E}}$ and satisfies System (14). Since ${\mathbf {x}}$ takes values from ${\mathcal {E}}$, we have ${\dot{x}}_{i,j}=z_j^*{\dot{q}}_i$. Since ${\mathbf {x}}$ also satisfies System (14), we have

$$\begin{aligned} {\dot{x}}_{i,j}=x_{i,j}\left( {\mathbf {T}}{\mathbf {p}}_{j}\right) _i-\left( {\mathbf {B}}{\mathcal {K}}{\mathbf {B}}^{\top }\left( \frac{{\mathbf {r}}_{i}^{*}}{{\mathbf {z}}^{*}}\right) \right) _{j}=q_i{z_j^*}^2(\mathbf {Tq})_i-q_i\left( {\mathbf {B}}{\mathcal {K}}{\mathbf {B}}^{\top }\mathbb {1}^m\right) _j=q_i{z_j^*}^2(\mathbf {Tq})_i. \end{aligned}$$

Thus, $z_j^*{\dot{q}}_i=q_i{z_j^*}^2(\mathbf {Tq})_i$ which implies that

21 $$\begin{aligned} {\dot{q}}_i=z_j^*q_i(\mathbf {Tq})_i, \end{aligned}$$

for $i=1,\ldots ,n$ and $j=1,\ldots ,m$. We now consider two cases.

**Case 1: The model is heterogeneous, i.e., the vector** ${\mathbf {z}}^*$ **is not parallel to** $\mathbb {1}^m$.

In this case, Eq. (21) will be satisfied only if $q_i(\mathbf {Tq})_i=0$ for $i=1,\ldots ,n$. Since $q_i\in {\mathbb {R}}_+$ for $i=1,\ldots ,n$, it follows that $\mathbf {Tq}={\mathbf {0}}^n$. Since $(\mathbb {1}^n)^{\top }{\mathbf {q}}=1$, we have ${\mathbf {q}}={\mathbf {y}}^*$. This implies that $x_{i,j}=y_i^*z_j^*=x_{i,j}^*$ for $i=1,\ldots ,n$ and $j= 1,\ldots ,m$. Thus, the largest subset of ${\mathcal {E}}$ that is positively invariant w.r.t. System (14) consists of just the unique equilibrium ${\mathbf {x}}^*\in S^{mn}_{+}$. By LaSalle’s invariance principle, it follows that the equilibrium ${\mathbf {x}}^*$ is locally asymptotically stable w.r.t. all initial conditions in $S^{mn}_{+}$ in the neighbourhood of ${\mathbf {x}}^*$, and globally asymptotically stable w.r.t. all initial conditions in $S^{mn}_{+}$ provided that System (14) is persistent.

**Case 2: The model is homogeneous, i.e.** ${\mathbf {z}}^*=\frac{1}{m}\mathbb {1}^m$

In this case, Eq. (21) takes the form ${\dot{q}}_i=\frac{q_i}{m}(\mathbf {Tq})_i$. We have $x_{i,j}=q_iz_j^*=\frac{q_i}{m}$ and

$$\begin{aligned} {\dot{x}}_{i,j}=\frac{{\dot{q}}_i}{m}=\frac{q_i}{m^2}(\mathbf {Tq})_i=x_{i,j}({\mathbf {T}}{\mathbf {p}}_{j})_i. \end{aligned}$$

Consequently, the largest subset of ${\mathcal {E}}$ that is positively invariant w.r.t. System (14) consists of all vectors ${\mathbf {x}}(t)\in \, S^{mn}_{+}$ satisfying ${\dot{x}}_{i,j}=x_{i,j}({\mathbf {T}}{\mathbf {p}}_{j})_i$ with $x_{i,j}=x_{i,k}$ for $i=1,\ldots ,n$ and $j,k=1,\ldots ,m$. The proof for Case 2 again follows from LaSalle’s invariance principle. $\square$

**Figure 5:**
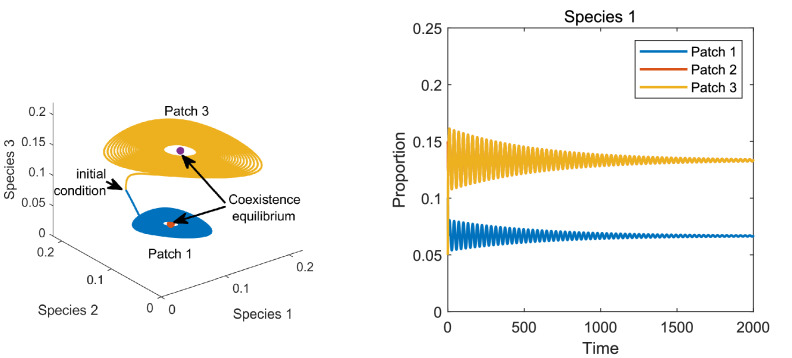
Left: Dynamics of the metapopulation model in Fig. 3 for patches 1 and 3 showing asymptotic stability of the coexistence equilibrium. Right: The time evolution of the proportion of species 1 in the three patches.

**Figure 6:**
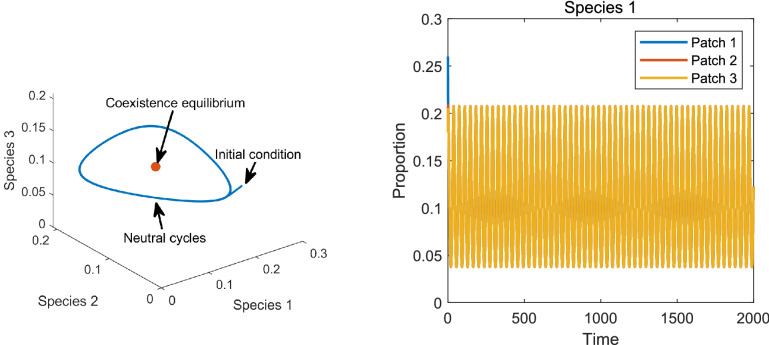
Left: Dynamics of the metapopulation model in Fig. 4 for patches 1 and 3 showing a limit cycle arising from the neutral stability of the coexistence equilibrium. Right: Time evolution of the proportion of species 1 in the three patches. Note that the dynamics in all patches are the same and thus the three graphs overlap.

The above results can be illustrated by simulating System (14) for the metapopulation models shown in Fig. 3 and 4 in Examples 1 and 2, respectively. The results of the simulations are shown in Figs. 5 and 6, respectively.

## Discussion and conclusion

In this paper, we have expanded the class of competition networks for which the numerical observations of Nagatani et al.^35^ are valid. This has been done, firstly, by giving a more general definition of homogeneity/heterogeneity of a metapopulation model that is based on the nature of the adjacency matrix of its inter-patch migrations rather than on the number of dispersal links as was previously done in^35^. Secondly, we have performed a stability analysis of the coexistence equilibrium of a metapopulation model whose inter-patch migration is assumed to be detailed balanced and whose intra-patch dynamics is governed by a mean-field ODE system with a coexistence equilibrium. Detailed balancing, a well-known concept in chemical reaction network theory (CRNT), is a key feature of many metapopulation models and previous studies had not explicitly explored this concept. The motivation behind using concepts from CRNT is based on the fact that much of the interesting dynamical behavior observed in biological systems can be understood by analyzing the underlying chemical components and CRNT provides a unified mathematical approach to the study of chemical processes. The assumption of detailed balancing thus allows us to view the inter-patch migrations as a detailed balanced single species mass action reaction network and make use of the already available results on the latter.

The results show that the considered metapopulation model admits a unique coexistence equilibrium. By making use of the Lyapunov function constructed in^46^, coupled with LaSalle’s invariance principle, it is shown that: (1) if the model is heterogeneous, then the coexistence equilibrium is locally asymptotically stable; it is globally stable if the considered metapopulation is persistent; (2) if the model is homogeneous and persistent, then the dynamics of the model is analogous to that of a single well-mixed patch; in this case, the coexistence equilibrium is neutrally stable. These results provide a mathematical support for the numerical results of^35^ and demonstrate that the numerical observations extend beyond the three-species cyclic systems to a larger class of networks.

It should, however, be noted that, as in^35^ and most metapopulation models, the above results have been achieved by examining the simplified case in which the patches are assumed to be homogeneous and contain a local population in which individuals are well mixed. In addition, it is assumed that all the patches contain the same species. Although this assumption is far from real metapopulations, it makes the mathematical analysis tractable and provides a starting point for future analysis of more realistic metapopulation models. However, the results reaffirm the intuition held by many community ecologists that spatial heterogeneities in the landscape can have profound effects on the dynamics of populations within an environment. In the metapopulation framework, these heterogeneities come in many forms ranging from differences in dispersal rates among the patches to patch size distributions, among others. Also the fact that fragmented habitats are more likely to present some level of spatial heterogeneity underlies the importance of the metapopulation framework in studies for nature conservation.
